# Effects of Replicative Senescence of Human Chorionic MSCs on their EV-miRNA Profile

**DOI:** 10.1007/s12015-024-10790-8

**Published:** 2024-09-21

**Authors:** Hedviga Košuthová, Lívia K. Fecskeová, Jana Matejová, Lucia Slovinská, Marko Morávek, Zuzana Bártová, Denisa Harvanová

**Affiliations:** 1 Associated Tissue Bank, Faculty of Medicine, Pavol Jozef Safarik University and Louis Pasteur University Hospital, Trieda SNP 1, 04011 Kosice, Slovakia; 2 Institute of Geotechnics of the Slovak Academy of Sciences, Watsonova 45, 040 01 Kosice, Slovakia

**Keywords:** Chorionic mesenchymal stromal cells, Extracellular vesicles, miRNA, Replicative senescence

## Abstract

Chorionic mesenchymal stromal cells (CHO-MSCs) and their extracellular vesicles (EVs) are becoming increasingly popular, since chorion is ethically harmless and an easily accessible source of MSCs. However, until now there is only a limited number of studies with a thorough characterization of CHO-MSCs derived EVs and their miRNA profile. In this study, we monitored changes in the EV-miRNA profile between early and late passage of human CHO-MSCs. First, senescence of CHO-MSCs was induced by serial passaging and confirmed by morphological changes, shortened telomeres and changes in the expression of selected genes. The expression of MSCs-specific surface markers CD73, CD90, CD105 did not change with increasing passages. Next, EVs and their miRNA profiles were compared between early vs late passage cells. Number of EVs and their size were not significantly changed. Seven of the top 10 most expressed EV-miRNAs were common to both early and late passages. A differential expression study between early and late passages identified 37 significantly differentially expressed EV-miRNAs, out of which 23 were found to be associated with pathways of cellular senescence based on KEGG pathway analysis. A set of 9 miRNAs were identified as the most frequently associated with senescence and/or with the most altered expression between early and late passages, out of which miR-145-5p, miR-335-5p and miR-199b-3p were the most significant downregulated miRNAs in late passages. The most upregulated EV-miRNAs were miR-1307-3p, miR-3615 and miR320b. Targeting these miRNAs in future experiments may prolong the therapeutic potential of CHO-MSCs and their EVs.

**Graphical Abstract:**

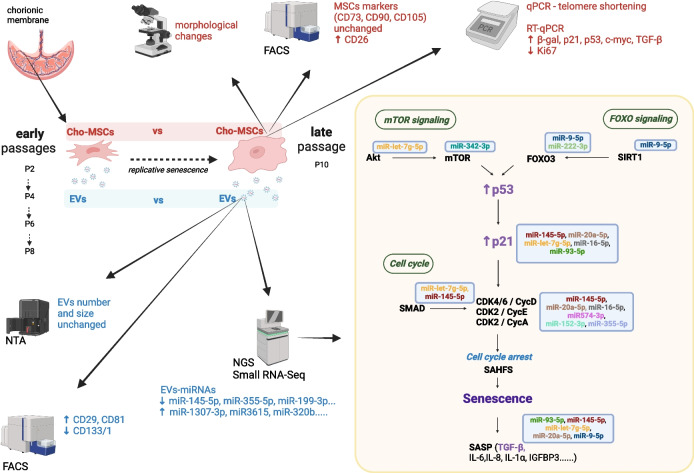

**Supplementary Information:**

The online version contains supplementary material available at 10.1007/s12015-024-10790-8.

## Introduction

Mesenchymal stromal cells (MSCs) are multipotent cells found in almost all types of tissues, they play an essential role in maintaining tissue and organ homeostasis *in vivo*. MSCs have a long-term ability of self-renewal and extensive differentiation potential, possess homing and trophic properties modulating the immune system, influencing microenvironment and enhancing tissue repair. This made MSCs a useful tool in a wide range of clinical applications, in regenerative medicine, tissue engineering and immunotherapy [1–3]. The ISCT (The International Society for Cell & Gene Therapy) committee issued minimal criteria to define multipotent MSCs as being plastic adherent, expressing CD73, CD90 and CD105, lacking the expression of hematopoietic and endothelial markers CD11b, CD14, CD19, CD34, CD45, CD79a and HLA-DR and capable of *in vitro* differentiation into adipocyte, chondrocyte and osteoblast lineages [4]. However, MSCs isolated from various tissues, even if they meet ISCT criteria, differ in many ways in terms of gene expression, differentiation potential and other characteristics which depend also on the age of the donor and the number of cell culture passages. The ISCT committee continues to support the use of the acronym “MSCs” but recommends be (i) supplemented by tissue-source origin of the cells, which would highlight tissue-specific properties; (ii) intended as MSCs unless rigorous evidence for stemness exists that can be supported by both *in vitro* and *in vivo* data; and (iii) associated with robust matrix of functional assays to demonstrate MSCs properties, which are not generically defined but informative for the intended treatment regimen and effect [5].

Although the bone marrow MSCs are still the gold standard, the use of MSCs from chorion is increasingly popular because of the absence of invasive procedures or ethical issues [6, 7]. CHO-MSCs show superior differentiation, immunosuppressive, and angiogenic potentials in comparison with haploidentical maternal placental cells [8]. Moreover, comprehensive characterization of CHO-MSCs showed reduced telomere loss and delayed onset of cellular senescence *in vitro* compared to MSCs from bone marrow, adipose tissue, and umbilical cord [9].

The yield of MSCs from tissues is very low. Therefore, their multiplication under *in vitro* conditions is essential for their use in research and clinical practice. However, repeated passaging carries the risk of replicative senescence [10, 11], which is associated with morphological changes, affecting the division capacity and differentiation potential of MSCs, reducing their efficiency, shortening telomeres, changes at the level of the transcriptome, phenotype and ultimately can lead to malignant transformation [12–14]. *In vitro* senescence can be proved at the transcriptome level as downregulation of proliferation marker Ki67 (gene MKI67) [15] together with increased level of β-galactosidase (gene GLB1), and components of the p53 pathway (for example p53, p16Ink4a and/or p21) [16, 17].

Recent investigations have revealed that the therapeutic effects of MSCs are largely mediated by the secretion of paracrine factors, mainly by extracellular vesicles (EVs) [18, 19]. The International Society for Extracellular Vesicles (ISEV) defined EVs as “the generic term for particles naturally released from the cell that are delimited by a lipid bilayer and cannot replicate, i.e. do not contain a functional nucleus” [20, 21]. Research outcomes of the last decade suggest that therapeutic use of MSCs-derived agents might be more promising than the use of intact MSCs. EVs are overcoming several limitations and practical challenges observed in cell-based therapy, [18, 19]. EVs transfer functional cargos like noncoding RNA species (ncRNAs), mRNA, DNA, peptides, proteins, cytokines and lipids from MSCs to the recipient cells. Among ncRNAs, the most attention is focused on microRNAs (miRNAs), single stranded non-coding RNAs transcribed from DNA, composed of about 22 nucleotides [22, 23]. MicroRNAs are a form of epigenetic regulators that alter the gene expression of a targeted mRNA without changing the genetic code. They regulate gene expression at the post-transcriptional level by binding to the 3´-untranslated regions of target mRNAs. MicroRNAs are involved in intercellular communication, they play a role in every cellular process, whether related to development, differentiation or maintenance of homeostasis. Since the expression of miRNAs changes during the onset and progression of certain illnesses, they could be used as practical tools for diagnosis, follow up, management and monitoring of various diseases. However, the same miRNA can regulate the expression of different mRNAs, their role is very complex and sometimes difficult to interpret [24].

Aging and replicative senescence of MSCs could cause changes in the size, number, and composition of their EVs, and expressions pattern of different miRNAs [25]. Emerging evidence suggests that EVs, at least partially by providing certain miRNAs, participate in a complex cell senescence network. MiRNAs are implicated in not only contributing to but also being influenced by MSCs senescence. Several differentially expressed miRNAs with different mode of action have been identified in various type of senescent MSCs. However, different profiles of miRNAs expression changes in different types of MSCs, which is related to the existence of different mechanisms of aging in MSCs from various sources [26–29].

In this study we evaluated the effect of serial passages on EVs and EV-miRNA profile. Understanding MSCs senescence at the miRNA level could allow us to influence MSC cultures *in vitro* to grow for a longer time without signs of senescence and without reducing the therapeutic efficacy of MSCs cultures and their EVs.

## Methods and Materials

### Isolation and Cultivation of CHO-MSCs

The study was approved by the Ethics Committee of the P. J. Safarik University and L. Pasteur University Hospital in Kosice, Slovakia (Approval ID 2020/EK/09067) and was conducted in accordance with the declaration of Helsinki. All donors gave their informed consent for inclusion before they participated in the study. Placentas from healthy donor mothers (n=3) less than 30 years old, were collected after cesarean sections and placed into the transport medium (DMEM (Sigma Aldrich, Germany) with 80 µg/mL gentamicin (Sigma Aldrich, Germany), cut into small pieces (1.5×1.5 cm) and treated for 4 hours with 0.1% (v/v) bacterial collagenase type II (Gibco, USA) at 37 °C. After digestion, the pieces of tissues were filtered through a 40 µm cell strainer (Falcon, BD Biosciences, USA) and the filtrate was centrifuged (Hettich, Germany) at 300 × g for 7 min. The obtained chorionic cells were cultured in a complete cultivation medium containing α-MEM (Sigma Aldrich, Germany), supplemented with 10% FBS (Gibco, Netherlands), 1% (*v*/*v*) antibiotic/antimycotic solution and 1% L-glutamine (Sigma Aldrich, Germany). Cells were maintained in 75 cm^2^ culture flasks (Sarstedt AG & Co., Germany) at 37 °C, 95% humidity in a 5% CO_2_ atmosphere. The cultivation medium was changed twice a week. When the cells reached 80% confluence, they were detached from the flask by 0.05% Trypsin-EDTA solution (Gibco, Netherlands) and seeded at a density of 2 × 10^3^ cells/cm^2^. Cell culture was passaged every 15-20 days, when the cells reached 80% confluence. The cell number was assessed by a TC10^TM^Automated Cell Counter (Bio-Rad Laboratories, USA). The cells were expanded for 10 passages. Cells from the 2^nd^, 4^th^, 6^th^, 8^th^ and 10^th^ passages (P2, P4, P6, P8, P10) were used for immunophenotype characterization, DNA and RNA isolation. The morphology of CHO-MSCs was observed under a light microscope (DM IL LED Leica, USA).

### Preparation of CHO-MSCs Conditioned Medium

When CHO-MSCs reached 80% confluence, the cells were carefully washed in Phosphate buffer solution (PBS) and incubated in DMEM (Sigma Aldrich, Steinheim, Germany) for 24 h at 37 °C in a humidified atmosphere and 5% of CO_2._ Culture conditioned media (CCM) were collected, centrifuged at 300 g for 7 min at 4 °C, filtered through 0.22 µm filter, concentrated 5 times using Amicon® Stirred Cells with Ultracel®3 kDa Ultrafiltration Discs (Merck Life Science, USA) and stored at −80 °C until use. CCM was used for EVs and miRNAs isolation.

### Immunophenotype Characterization of CHO-MSCs

Immunophenotype characterization of CHO-MSCs was performed by flow cytometry. At least 2 × 10^5^ (cells/mL) CHO-MSCs from P2, P4, P6, P8 and P10 were resuspended in 0.1 ml PBS (Sigma Aldrich, Germany) containing 2 % FBS (Sigma Aldrich, USA). The cell suspension was incubated with anti-human monoclonal antibodies: CD90, CD105, CD73, CD55, CD54, CD26, CD45, CD34 and HLA-DR (Miltenyi Biotec GmbH, Germany) for 10 min at room temperature. MSCs surface markers were analyzed by a Becton Dickinson FACSCalibur using CellQuest software (Becton Dickinson, USA). Differences in cell surface markers expression in the early and late passages were compared by a t-test.

### DNA Isolation and Telomere Length Analysis

DNA of CHO-MSCs was isolated from approximately 1×10^6^ cells/mL from P2, P4, P6, P8 and P10. Cell pellets were prepared by centrifugation (Hettich, Germany) of cells suspension at 1500 × g for 7 min at 4 °C. After removing the supernatant, the pellets were stored at -80°C until isolation. Prior to DNA isolation, cells were slowly thawed on ice and loosened by flicking the tube several times. The cell pellets were then resuspended in 100 µl cold 1× PBS (Sigma Aldrich, Germany) by pipetting up and down. The resulting suspension was used for DNA isolation using the Monarch® Genomic DNA Purification Kit (NEB, USA) according to the manufacturer’s instructions. Briefly, 1 µl Proteinase K and 3 µl RNase A were added to samples and vortex. Then was added 100 µl of Cell Lysis Buffer. Samples were again vortex and incubated 5 min at 56 °C in a thermal mixer Multitherm (Benchmark Scientific, USA) with agitation in full speed. After this, 400 µl of gDNA Binding Buffer was added and samples were transfer to purification column pre-inserted into a collection tube. First, samples were centrifuged for 3 minutes at 1000 × g to bind the gDNA and then for 1 min at ≥ 12 000 × g to clear the membrane. Then was the membrane twice washing by 500 µl Wash Buffer and centrifuge for 1 min at ≥ 12 000 × g. Elution was performed by 60 µl of preheated (60 °C) Elution Buffer and centrifugation for 1 min at ≥ 12 000 × g. Concentration and purity of the samples was determined using NanoPhotometer^®^ P330 (Implen, Germany). For analysis of absolute telomere length, 5 ng of DNA from each sample was used in the Absolute Human Telomere Length Quantification qPCR Assay Kit (ScienCell^TM^ Research Laboratories, CA, USA). PCR preparation and telomere length calculation were performed according to the manufacturer’s protocol. The real-time PCR reaction was run using the following thermal cycling program: initial denaturation 95°C, 10 min; 32 cycles (denaturation 95 °C, 20 s; annealing 52 °C, 20 s; extension 72 °C, 45 s; plate read), melting curve analysis. All samples were prepared in triplicates. Differences in telomere length between the early and late passages were compared by a non-parametric, unpaired t-test.

### RNA Isolation and Analysis of Gene Expression by RT-qPCR

Total RNA of CHO-MSCs was isolated from approximately 1×10^6^ cells/mL from P2, P4, P6, P8 and P10. Cell pellets were prepared by centrifugation of cells suspension at 1500 × g for 7 min at 4 °C. Immediately were lysed with 1 ml of TRIzol reagent (Ambion, USA) and stored at -80°C. Before isolation 200 µl of chloroform in molecular biology grade (Serva, Germany) was added to thawed lysed cell suspension and centrifuged at 12 000 × g for 15 minutes at 4 °C. Total RNA was isolated from aqueous phase using the Monarch® Total RNA Miniprep Kit (NEB, USA) according to the manufacturer’s protocol for TRIzol – extracted samples. Briefly, the aqueous phase was transferred to new tube, mixed with equal volume of ≥ 95 % ethanol in molecular biology grade (Merck, Germany) and transferred to purification column. Then was membrane washing with 500 µl of Wash Buffer and spin for 30 s at 16 000 × g. DNase in DNase I Reaction Buffer was pipet directly to the top of membrane. After 15 min incubation 500 µl of Priming Buffer was added and sample were spined for 30 s at 16 000 × g. After twice wash with Wash Buffer and twice spin at 16 000 × g, the samples were eluted with 60 µl of nuclease-free water. Concentration and purity of the samples was determined using NanoPhotometer^®^ P330 (Implen, Germany). 1 µg of RNA was reverse transcribed using SuperScript™ IV First-Strand Synthesis System (Invitrogen, USA) using oligo(d)T primers, according to the manufacturer’s protocol. The obtained cDNA was diluted 10× and used directly in qPCR or stored at −80 °C. Quantitative real time PCR (qPCR) was performed using PowerUp™ SYBR™ Green Master Mix (Applied Biosystems, USA) on a CFX96 Real-Time Detection System (BioRad, USA). A 10 μL reaction contained 1× PowerUp Sybr Green master mix, 200 nM of each forward and reverse primer and 1.5 μL of 10 × diluted cDNA. Relative gene expression of the selected target genes was calculated using the 2^-ΔΔCt^ method [30]. Gene expression was normalized to the HPRT reference gene. A set of reference genes GAPDH, YWHAZ, RPL13, HPRT, β-actin were previously tested using the geNorm algorithm [31] and HPRT was selected as the most stable gene. The list of selected target genes and their primer sequences are available in Table 1. All samples were prepared in triplicates. Differences in gene expression in the early and late passages were compared by a non-parametric, unpaired t-test.

**Table 1:** The list of primers used in RT-qPCR

Gene (Target)	Forward primer sequence (5´- 3´)	Reverse primer sequence (5´- 3´)
HPRT	CATTATGCTGAGGATTTGGAAAGG	CTTGAGCACACAGAGGGCTACA
GLB (β - gal)	CACTCCACAATCAAGACCGAAGC	CTGTGCTGCATAGGGTGAGTTG
MKI67 (Ki67)	GAAAGAGTGGCAACCTGCCTTC	GCACCAAGTTTTACTACATCTGCC
CDKN1A (p21)	GGACAGCAGAGGAAGACCATGT	CGGCGTTTGGAGTGGTAGAA
TP53 (p53)	CCTCAGCATCTTATCCGAGTGG	TGGATGGTGGTACAGTCAGAGC
c-myc	CCACAGCAAACCTCCTCACA	CGGTTGTTGCTGATCTGTCTCA
E-Ras	GCAAGAGTGCGCTGACCAT	GCCCAGCACACCATCACA
TGF - β2	AAGAAGCGTGCTTTGGATGCGG	ATGCTCCAGCACAGAAGTTGGC

### EVs Isolation

For the isolation of EVs and EV-miRNAs, 5× concentrated CCM were used. EVs were isolated using ExoQuick-TC precipitation reagent (System Biosciences, USA). Specifically, CCM were firstly centrifuged at 3000 × g, for 15 min at RT. Consequently, 1 mL of the reagent was added to 5 mL of CCM, thoroughly mixed, and incubated overnight at 4°C. After incubation, samples were centrifuged at 1500 × g, 30 min at RT. Pellets containing EVs were resuspended in PBS and stored at -80°C.

### Nanoparticle Tracking Analysis

Size distribution and concentration of EVs were analyzed by Nanoparticle Tracking Analysis (NTA) on LM10B Nanoparticle Characterization System (Nano Sight, UK) with a trinocular microscope and LM12 viewing unit with a 60mW laser working at λ = 405 nm. Samples were filtered through a 0.22 µm filter before measurement. Results were displayed as a number-weighted particle size distribution. Video sequence was recorded via CCD camera operating at 30 frames per second and evaluated through the NANOSIGHT NTA 3.4 Analytical Software Suite.

### Multiplex Bead-Based Flow Cytometry Assay

Analysis of surface protein expression on EVs (early vs late passages) was performed using the MACSPlex Exosome human kit (Miltenyi Biotec, Germany) following the manufacturers protocol for overnight incubation. This kit enables the detection of 37 markers (CD1c, CD2, CD3, CD4, CD8, CD9, CD11c, CD14, CD19, CD20, CD24, CD25, CD29, CD31, CD40, CD41b, CD42a, CD44, CD45, CD49e, CD56, CD62p, CD63, CD69, CD81, CD86, CD105, CD133.1, CD142, CD146, CD209, CD326, HLA-ABC, HLA-DR DP DQ, MCSP, ROR1 and SSEA-4) simultaneously and include the two isotype controls (mIgG1 and REA control). Before analysis, EVs were isolated by ultrafiltration using Amicon Ultra Centrifugal Filter Devices. 120 µl of isolated EVs or MACSPlex buffer as blank control (both in triplicates) were incubated with 15 μl capture beads overnight at RT under gentle shaking (450 rpm) and protected from light. The EV-bead complexes were washed and centrifuged at 3000 × g for 5 min at RT. Detection antibody mixture (CD9, CD63 and CD81 conjugated to APC) was added to the beads, samples were mixed by gentle vortexing and incubated for 1 h at RT under gentle shaking (450 rpm) and protected from light. The samples were washed two times before analyzed on a BD FACSCalibur using CellQuest software (BD Biosciences). Each EV marker’s median fluorescence intensity (MFI) was normalized to the mean MFI for specific EV markers (CD9, CD63, and CD81) after the values of the control (MACSPlex buffer) were subtracted from all samples.

### EV-miRNA Isolation and Small RNA Sequencing

EV-miRNAs were isolated from 5× concentrated CCM. EVs small RNA (including miRNA) was isolated using the SeraMir Exosome RNA Purification kit (System Biosciences, USA) according to the manufacturer´s protocol. The quality and quantity of small RNA was inspected with Bioanalyzer (Agilent, USA) using Small RNA kit (Agilent, USA). Consequently, RNA was sent to Novogene (UK) for Small RNA-Seq on Illumina platform in 1×50 bp reads. Bioinformatic analysis of raw reads up to mapping to the human genome was provided by Novogene. Due to the relatively costly nature of the analysis, sample size was reduced for Small RNA-Seq. Based on results from the cellular level, P4 and P6 were selected as early, and P10 as late passage samples. In total 9 samples were sequenced (early n=6, late n=3), however due to insufficient amount of sample, sequencing of 2 samples resulted in very low data output and were excluded from the data analysis, resulting in 7 samples in total (early n=5 (P4, P6), late n=2 (P10)).

### Differentially Expressed miRNAs, Target Search and Pathway Analysis

Differentially expressed (DE) miRNAs between late vs early passages were analyzed by the DeSeq2 package v1.32 [32] using fittype ‘parametric’ in RStudio v4.1.1. For each miRNA, significant p-values and false discovery rates (FDR) adjusted p-values (p-adj) were obtained. The criteria for DE-miRNA have been set as log_2_ fold change >1 and p-adj < 0.05. Heatmap of DE miRNAs were created using the pheatmap R package v1.0.12 [33] using log2 TPM values and euclidean distance for clustering. Potential gene targets of DE miRNAs were searched in the mirTarBase database of experimentally validated miRNA-mRNA interactions, release 9.0 [34]. Only targets verified by Western blot, qPCR and reporter assay were considered, since these represent strong experimental evidence. Next, Gene Ontology enrichment analysis was performed for miRNA targets at the GO website (https://geneontology.org/docs/go-enrichment-analysis/) using the PANTHER Classification System (release 18.0) for cellular components, molecular functions and PANTHER pathways terms [35]. A KEGG enrichment analysis was performed for miRNA targets, as well, using ShinyGO 0.80 online platform [36] at http://bioinformatics.sdstate.edu/go/. KEGG pathways related to cellular senescence were selected and protein targets assigned to these pathways were used to identify miRNAs related to cellular senescence. Plots were created using the ggplot2 R package, v3.4.4 [37].

## Results

### Cellular Characteristics of Senescence

#### Morphology of CHO-MSCs

**Fig. 1:**
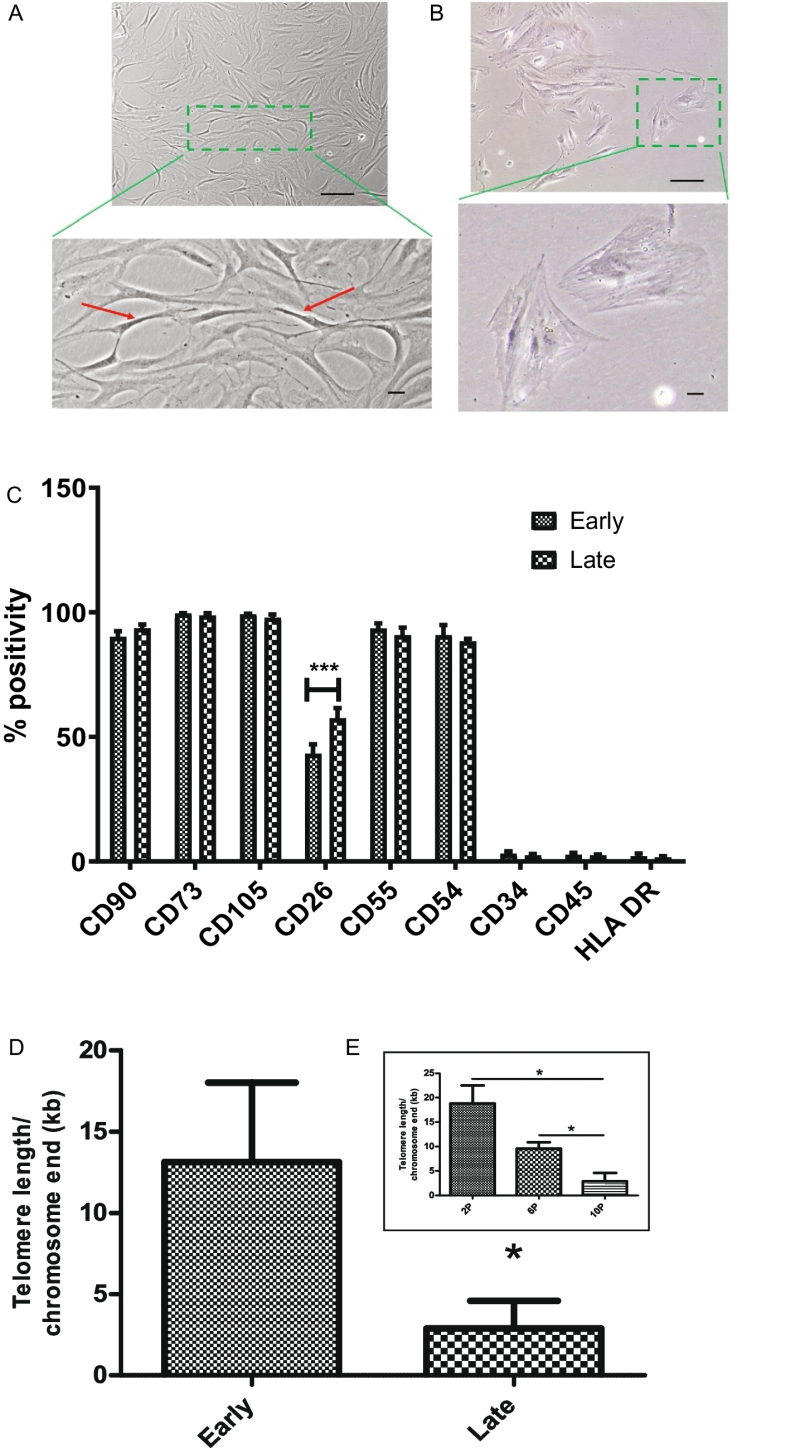
Changes in CHO-MSCs over serial passaging at the cellular level: **A**, **B** Phase-contrast images and corresponding details (boxed areas) of human CHO-MSCs. CHO-MSCs changed morphology during prolonged *in vitro* culture. The picture shows cells of P4 (A) and P10 (B). Scale bar = 200 μm, 50μm (higher magnification of boxed area). **C** Bar chart of flow cytometry data showing cell surface marker expression of CHO-MSCs at early (P2-P8) vs. late (P10) passages, n=3. Values are shown as percentage positive expression of total cells analyzed ± SD. *p < 0.05; **p < 0.01; ***p < 0.001. **D**, **E** Comparison of the average telomere length per chromosome end in the early (P2-P8) vs late (P10) passage CHO-MSCs (D) and their gradual shortening in the P2, P6 and P10 (E), shown as the mean ±SD. *p < 0.05; **p < 0.01; ***p < 0.001

Early passages of CHO-MSCs (P2, P4, P6, P8) showed elongated, fibroblastic-like, spindle-shape morphology with parallel orientation at confluence (Fig. 1A). Cells were exhibiting this phenotype up to P8. After P8, the cells started to undergo morphological changes, they increased in size and began to acquire a flat shape (Fig. 1B). This change was accompanied also by decreased proliferation. The late passage (P10) cells did not cover 80% of the surface of the culture flask even after 3 weeks of cultivation, while 2 weeks were sufficient for early passages.

#### Immunophenotype Characterization

The characterization of possible changes in CHO-MSCs’ immunophenotype of early and late passages was performed by flow cytometry. The percentage of positivity of each marker in early (P2-P8) and late passages (P10) is shown in Fig. 1C and in every second passage individually in the Supplementary Figure S1. Flow cytometry analysis indicated that CHO-MSCs at various passages were highly positive for MSCs specific surface markers (CD73, CD90, CD105) and these expressions did not change with increasing passages. The expressions of CD54 and CD55 were also stable during serial passaging. On the other hand, the expression of CD34, CD45 and HLA DR markers were negative at all tested passages. Statistically significant changes were observed only for the marker CD26, whose expression increased with increasing passage.

#### DNA Isolation and Telomere Length Analysis

Telomere length is an important indicator of cell senescence. The average telomere length on each chromosome end was 13.14 ± 0.96 kb for early passage CHO-MSCs versus 2.88 ± 0.21 kb for late passage with a statistical significance (p<0.05; Fig. 1D), however a gradual shortening of telomeres was observed towards increasing passage numbers (Fig. 1E).

#### Gene Expression Analysis by RT-qPCR

**Fig. 2:**
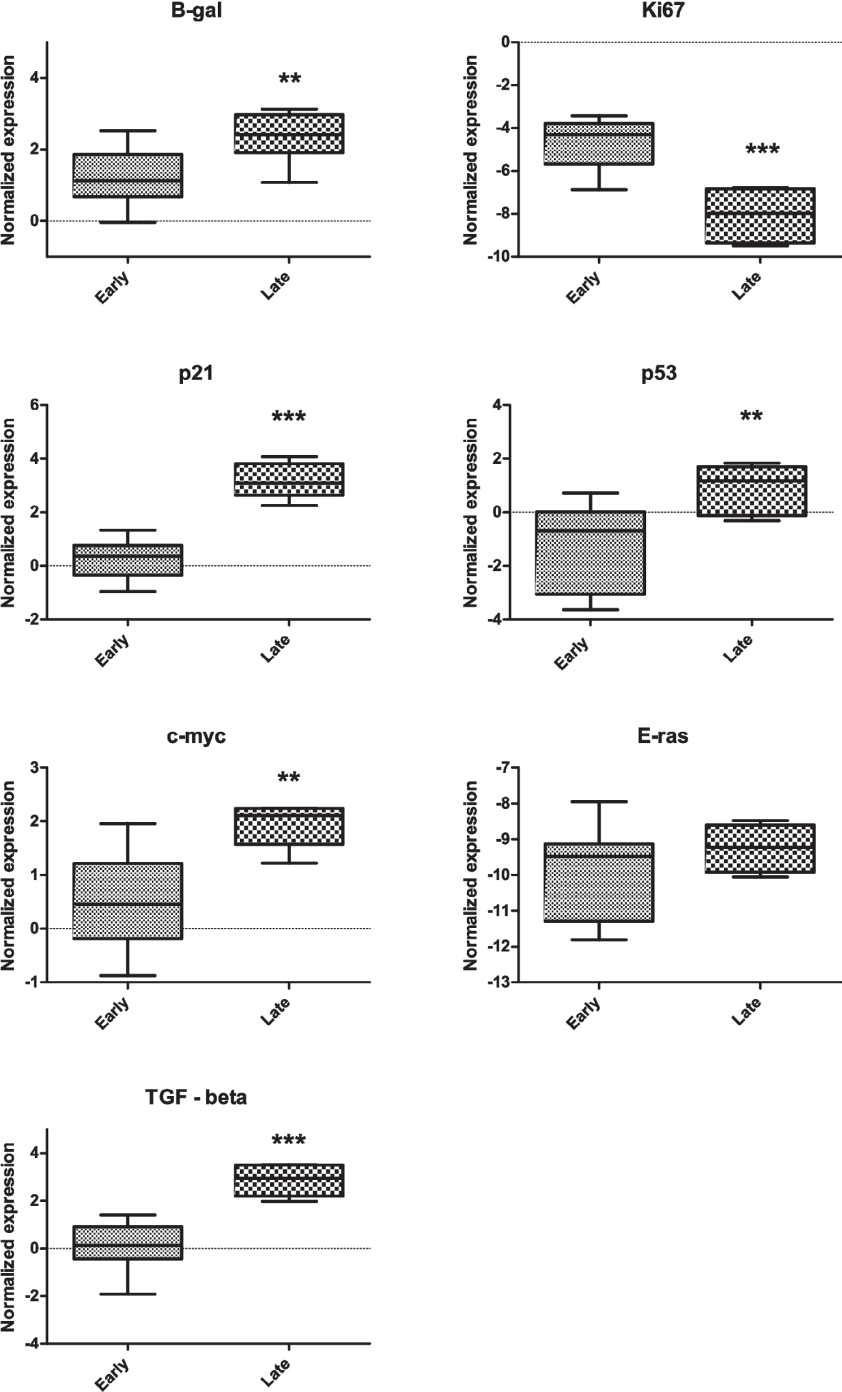
Comparison of relative expression of senescence related genes p21, p53 and β-gal, proliferation marker Ki67, oncogenes c-myc and E-ras and TGF-β between early (P2-P8) and late (P10) passages of CHO-MSCs. Gene expression is shown as normalized expression (-ΔCt, normalized Ct). Groups were compared by non-parametric, unpaired t-test, significance shown as * p < 0.05; ** p < 0.01; *** p < 0.001

We examined changes in gene expression of CHO-MSCs after serial passages (Fig. 2, Supplementary Fig S2) and based on the relative expression of selected genes we confirmed the senescence status of the P10. Expression of the two hallmark genes of senescence p21 and p53 were significantly upregulated in this passage. The upregulation was observed also in the senescence marker β-galactosidase. Together with the downregulation of the proliferation marker MKI67 (Ki-67), this demonstrates the senescence of cells in P10. E-Ras and c-myc belong to proto-oncogenes. While E-Ras gene expression did not change significantly with increasing passage number, c-myc expression significantly increased in the late passage. Significant changes in gene expression were also observed in TGF-β1, which was upregulated in the late passage. Products of this gene are involved in multiple biological processes. In relation to repeated passage and aging, their role is primarily associated with senescence and suppressing several proliferation factors.

### Extracellular Characteristics of Senescence

Studying the markers of senescence in individual passages of CHO-MSCs, results showed that senescence appeared suddenly at the 10^th^ passage. Apart from the gradual shortening of the telomeres and the increasing expression of CD26 through the passages, cells displayed a similar phenotype (Fig. 1A, B, C; Suppl Fig S1) and gene expression pattern (Fig. 2, Suppl Fig S2) till the 8^th^ passage, hence all the passages from P2 to P8 were considered as early and P10 represented the late passage.

#### EVs

**Fig. 3:**
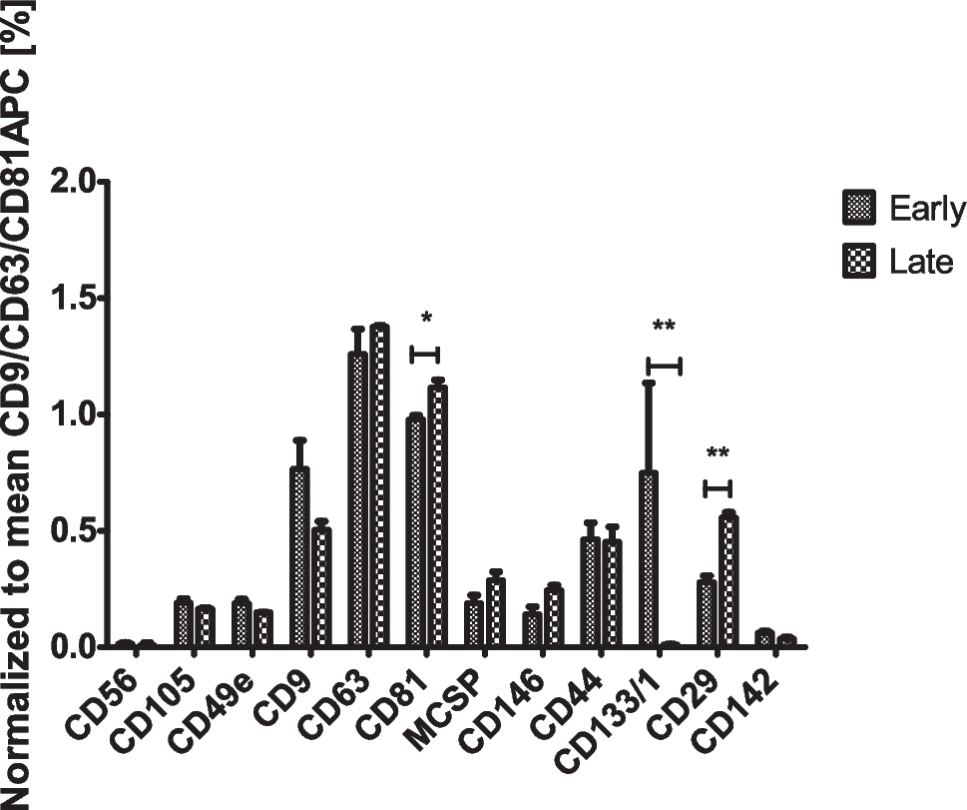
Surface marker profile of EVs isolated from early (P2-P8) and late (P10) passages. EV surface proteins were characterized using multiplex bead-based flow cytometry assay. Data is presented as CD9/CD63/CD81 normalized background subtracted (isotype control and blank samples) median fluorescence intensity (MFI). Data sets are presented as mean ± SD (n = 3). Groups were compared by non-parametric, unpaired t-test, significance shown as * p < 0.05; ** p < 0.01; *** p < 0.001

EVs isolated from CCM of early and late passages of CHO-MSCs were characterized in terms of size and number by NTA, and expression of EVs surface markers by flow cytometry. NTA analysis showed that average number of EVs normalized per number of cells and their size were not significantly changed between early and late passages (76.1±37.9 vs. 86.8±97.2, 191.2±18.6 nm vs. 206±20.3 nm, respectively). Flow cytometry analysis detected the expression of 10 surface markers out of 37, including the typical EVs surface markers CD81, CD9 and CD63. Expression of most of the markers did not change through the passages, however CD81 and CD29 significantly increased and CD133 significantly decreased towards the aging of the cells (Fig. 3).

#### EV-miRNAs

Small RNA from CHO-MSC derived EVs were isolated from CCM and evaluated on Bioanalyzer for quantity and quality. In total 7 samples of CHO-MSC-EVs small RNA from passages P4 & P6 (representing early passage samples) and P10 (representing late passage samples), originating from 3 biologically different chorionic tissues were sequenced. Since we did not observe significant differences between the early passage cells (P2-P8), passages P4 and P6 were chosen for sequencing based on the highest miRNA concentrations. Sequencing resulted in more than 88 million high quality reads with an average read of 12.63 ± 1.1 million per sample. 1030 known and 14 unknown miRNAs were identified, out of which 932 miRNAs were identified in the early, and 607 miRNAs in the late passage groups. Known miRNAs represented 1.38 - 10.26% of all vesicular small RNAs with no statistically significant difference between the early and late passage samples. Read counts of miRNAs were normalized by the method TPM for the comparison of individual samples and sample groups. Out of the top10 most abundant EV-miRNAs, 7 were common to both early and late passages: hsa-miR-21-5p, hsa-miR-143-3p, hsa-let-7i-5p, hsa-let-7b-5p, hsa-let-7f-5p, hsa-miR-26a-5p, hsa-let-7a-5p.

**Fig. 4:**
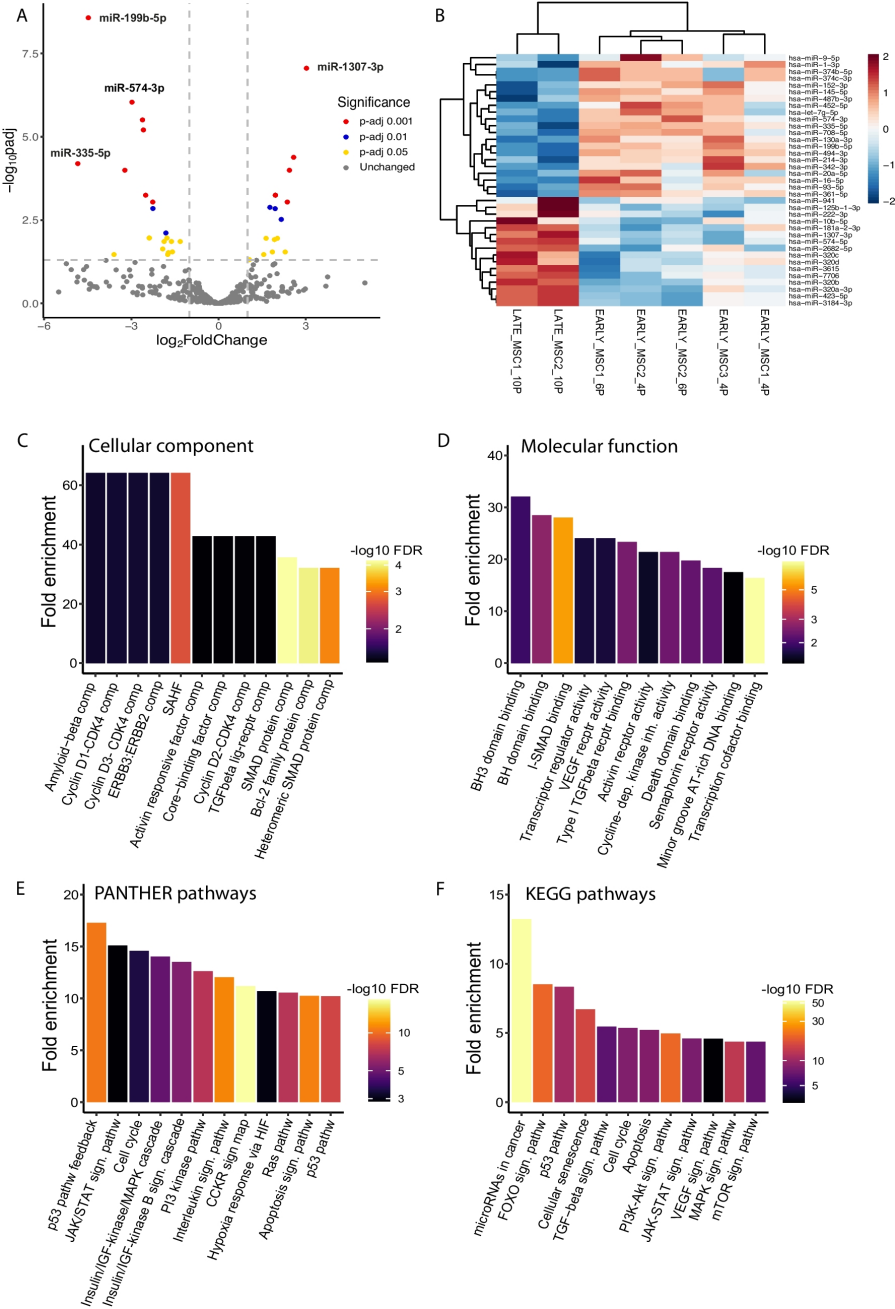
Results of the analysis of differentially expressed miRNAs. **A** Volcano plot of differentially expressed (DE) miRNas. The volcano plot shows log2 fold change on the x-axis and False Discovery Rate adjusted p value (p-adj) on the y-axis. Dots represent individual miRNAs shown by different color-coding according to the significance of the differential expression. log2 fold change >1 and < 1 shown as up- and down-regulated miRNAs, respectively. **B** Heatmap colored by scaled values of log2 TPM of DE miRNAs between early and late passages. Samples are clustered by euclidean distance. **C-F** Gene ontology analysis of cellular component (C) and molecular function (D) enrichment, and PANTHER (E) and KEGG (F) pathway analysis of DE miRNAs target genes. C, D, E show the top 12 enriched terms based on the highest fold enrichment value, F shows selected KEGG pathways related to cellular senescence. SAHF – senescence associated heterochromatin focus; comp – complex; pathw – pathway, sign – signaling

We identified 37 statistically significantly differentially expressed (DE) miRNAs between the early (n=5) and late (n=2) passages (p-adj<0.05):16 up-regulated and 21 down-regulated, out of which 13 were statistically strongly DE (p-adj<0.001; Fig. 4A, B). Potential mRNA targets of DE miRNAs were assigned based on the miRTarBase database, which is a database of experimentally validated miRNA-mRNA interactions. Out of 37 DE miRNAs, 30 miRNAs were found to have experimentally validated target(s) that resulted in 407 protein targets in total and 331 unique protein targets. DE miRNAs and the number of their protein targets is summarized in Tables 2 and 3.

**Table 2:** List of significantly upregulated miRNAs in the late vs early passages, and the number of their experimentally validated protein targets in the mirTarbase database. The list is ranked by p-adj. AVRG TPM = average transcript per million, FCH = fold change, no of exp val targets = number of experimentally validated targets

*Upregulated miRNA*	*AVRG TPM* *Early ± STDEV*	AVRG TPM *Late ± STDEV*	*log2FCH*	*p-adj*	*No of exp.* *val. targets*
*hsa-miR-1307-3p*	193.3±82.4	1644.6±889.3	3.021	9.22E-08	0
*hsa-miR-3615*	117.4±48.5	751.7±258.6	2.580	4.22E-05	0
*hsa-miR-320b*	159.5±74.1	866.9±200.6	2.434	1.03E-04	3
*hsa-miR-423-5p*	563.9±219.7	2236.7±303.0	1.954	5.84E-04	5
*hsa-miR-3184-3p*	563.0±218.6	2236.7±303.0	1.956	5.84E-04	0
*hsa-miR-181a-2-3p*	129.5±91.6	664.6±102.1	2.366	9.16E-04	0
*hsa-miR-125b-1-3p*	1209.5±262.9	4354.3±3006.5	1.766	1.35E-03	2
*hsa-miR-320a-3p*	1806.5±915.4	7171.1±852.8	1.949	1.47E-03	0
*hsa-miR-7706*	53.2±25.5	251.2±25.8	2.155	3.00E-03	0
*hsa-miR-222-3p*	3815.1±1301.1	12536.8±9083.0	1.633	1.13E-02	26
*hsa-miR-320c*	67.1±29.9	271.9±145.6	2.023	1.13E-02	1
*hsa-miR-2682-5p*	198.6±126.2	781.9±52.6	1.926	1.23E-02	1
*hsa-miR-941*	190.2±78.6	765.5±771.5	1.850	2.85E-02	1
*hsa-miR-320d*	23.6±14.9	115.4±78.5	2.293	2.85E-02	0
*hsa-miR-10b-5p*	12829.7±4585.7	36175.4±25971.3	1.553	3.38E-02	6
*hsa-miR-574-5p*	625.2±94.5	1318.1±44.5	1.060	4.96E-02	2

**Table 3:** List of significantly downregulated miRNAs in the late vs early passages, and the number of their experimentally validated protein targets in the mirTarbase database. The list is ranked by p-adj. AVRG TPM = average transcript per million, FCH = fold change, Exp val targets = number of experimentally validated targets

*Downregulated miRNA*	*AVRG TPM* *Early ±STDEV*	*AVRG TPM* *Late ± STDEV*	*log2FCH*	*p-adj*	*No of exp.* *val. targets*
*hsa-miR-199b-5p*	324.5±183.3	13.8±7.4	-4.48	2.74E-09	7
*hsa-miR-574-3p*	1880.2 ±993.3	242.3±22.1	-2.98	9.61E-07	7
*hsa-miR-145-5p*	2237.2±770.0	386.8±209.1	-2.61	3.25E-06	66
*hsa-miR-494-3p*	259.2±45.1	42.1±11.1	-2.58	6.32E-06	13
*hsa-miR-335-5p*	73.2±24.8	2.0±2.8	-4.84	6.28E-05	19
*hsa-miR-1-3p*	572.9±286.4	55.8±63.8	-3.22	1.03E-04	37
*hsa-miR-214-3p*	858.1±506.7	147.1±93.1	-2.51	5.84E-04	27
*hsa-miR-93-5p*	304.5±135.3	66.1±21.4	-2.26	9.16E-04	11
*hsa-miR-708-5p*	195.6±59.4	40.0±14.2	-2.26	1.43E-03	6
*hsa-miR-487b-3p*	170.3±28.3	51.9±29.5	-1.81	7.65E-03	6
*hsa-miR-374b-5p*	95.1±49.0	18.1±1.6	-2.38	1.08E-02	3
*hsa-miR-374c-3p*	95.1±49.0	18.1±1.6	-2.38	1.08E-02	0
*hsa-miR-130a-3p*	178.9±58.5	53.4±9.1	-1.77	1.08E-02	14
*hsa-miR-452-5p*	291.9±141.1	82.8±3.9	-1.87	1.42E-02	3
*hsa-let-7g-5p*	42615.4±15631.1	14057.7±2843.3	-1.62	1.42E-02	12
*hsa-miR-152-3p*	2765.6±571.0	1156.7±342.2	-1.32	1.42E-02	15
*hsa-miR-342-3p*	142.3±73.4	37.6±4.8	-1.92	2.31E-02	11
*hsa-miR-16-5p*	201.9±78.9	66.0±5.6	-1.58	2.85E-02	35
*hsa-miR-20a-5p*	229.1±102.6	71.7±16.6	-1.73	2.99E-02	32
*hsa-miR-9-5p*	44.5±65.2	3.6±2.0	-3.60	3.38E-02	33
*hsa-miR-361-5p*	83.4±18.4	25.5±6.3	-1.75	3.38E-02	3

To determine whether miRNAs and their potential targets are functionally involved in pathways associated with cellular senescence, GO functional analysis of cellular components and molecular functions was performed. Enriched terms were indeed associated with mechanisms of, or related to cellular senescence, apoptosis and cell death. Senescence-associated heterochromatin focus (SAHF) was the term with the highest fold enrichment value and statistical significance among the cellular components, followed by SMAD protein complex, Bcl-2 family complex and heteromeric SMAD protein complex as terms with the highest statistical significance (Fig. 4C). Similarly, terms enriched in molecular function analysis were mainly related to apoptosis (BH-domain binding), cell death and transcription activity regulation (Fig. 4D).

**Fig 5:**
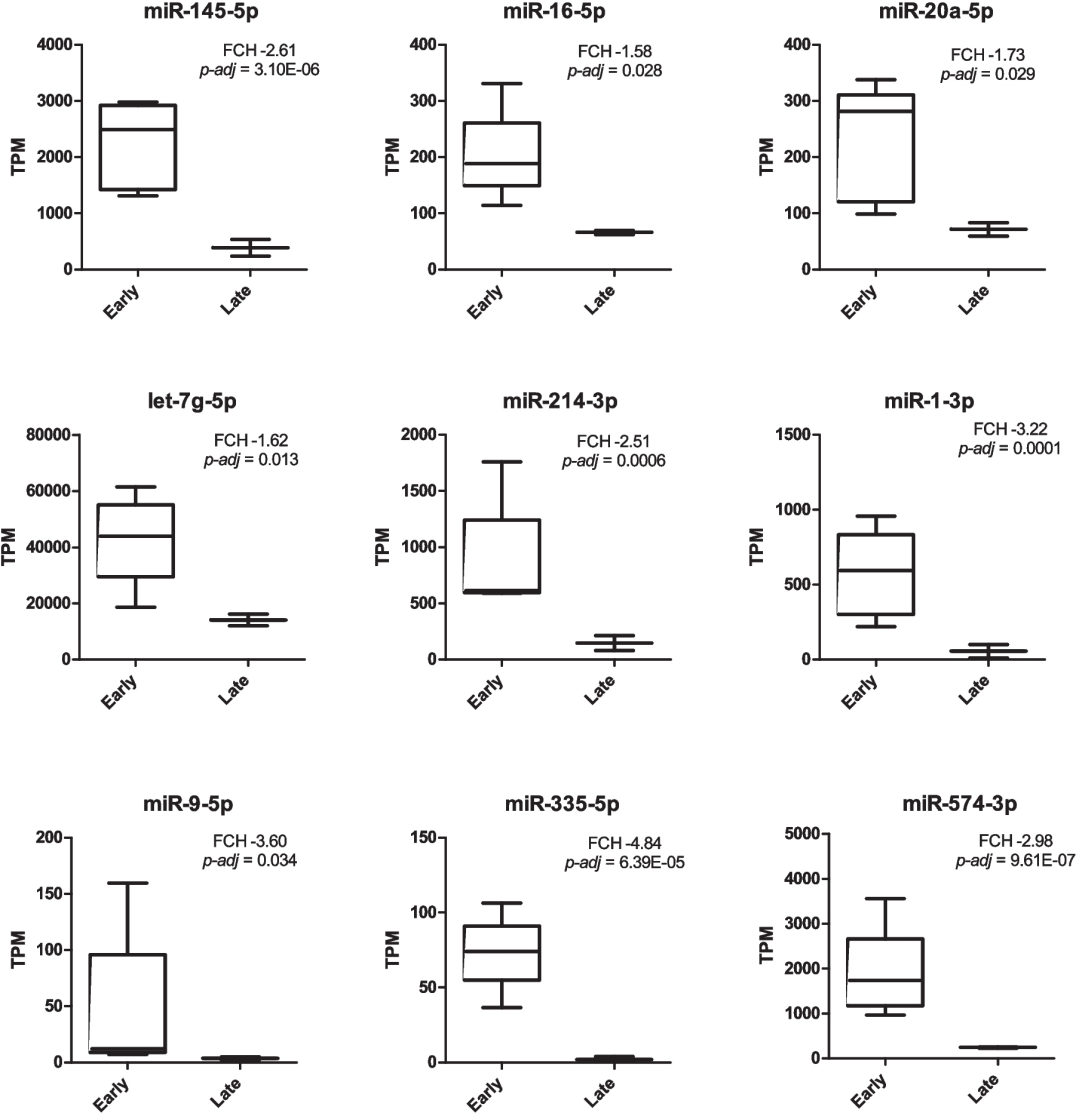
Expression levels of miRNAs most frequently identified in KEGG pathways associated with cellular senescence. Normalized expression is shown as TPM (transcript per million). Fold change (FCH) of downregulation and the p-adj value as determined by DESeq2 is shown

In pathway analysis, the p53, interleukin, CCKR and apoptosis signaling pathways had the highest statistical significance (Fig. 4E). A KEGG pathway analysis was performed, as well, and from the results, twelve pathways related to cellular senescence were selected (Fig. 4F). Out of these, microRNAs in cancer, FOXO and p53 signaling pathways were the most enriched, with 13.2-, 8.5- and 8.4-fold enrichment (FE), respectively (Suppl Tab S1). Pathways of cellular senescence (6.7 FE), TGF-β signaling pathway (5.5 FE), cell cycle (5.4 FE) and apoptosis (5.2 FE) followed as the most enriched selected KEGG pathways. Based on protein targets involved in these pathways and their associated DE miRNAs, we identified 9 miRNAs as the most frequently represented in the above-mentioned pathways and with the highest statistical significance of differential expression between the early and late passages (Suppl Tab S2). Potential targets of these miRNAs were overlapping in different pathways (Suppl Tab S1, S2) and most frequently were CDKN1A (p21), CDK4/6 and cyclins (CCNB, CCND, CCNE) related to cell cycle, kinases with complex cellular functions such as growth, proliferation, differentiation, survival, migration (PIK3CA, MAPK1 and AKT2), SMAD proteins related to TGF-β induced cell cycle arrest and apoptosis. Differential expression of these miRNAs and the value of log2 fold change is shown in Fig. 5.

## Discussion

In recent years, chorion has been used as one of the popular sources of MSCs because it does not require invasive procedures or controversial ethical issues [7]. However, an *in vitro* expansion process is required to obtain enough MSCs for research or therapy. Prolonged *in vitro* cultivations carry the risk of senescence and loss of therapeutic potential of MSCs. In this study, we used CHO-MSCs from young and healthy donors of age less than 30 years old. We were interested in the effect of serial passaging on the characteristics of CHO-MSCs, their EVs and EV-miRNA profile.

First, we induced replicative senescence in CHO-MSCs *in vitro* by serial passaging. Cells demonstrated characteristic immunophenotype of human MSCs according to ISCT minimal definition criteria [3]. The expression of typical MSC markers CD73, CD90, CD105 and markers CD54 and CD55 was stable during serial passaging (Fig. 1C). A statistically significant increase towards the late passages was observed for CD26 (Fig. 1C, Suppl Fig S1), which is associated with senescence, reduced immunopotency and overall loss of therapeutic effect compared to MSCs with normal levels of CD26 [38].

According to specific morphological changes (Fig. 1A, B), presence of significantly shorter telomeres (Fig. 1D, E) and increased expression of senescence-related genes (Fig. 2), we showed that chorion-derived MSCs from young, healthy donors reached senescence at passage 10. Previous studies have shown that depending on the source of MSCs, as well as the age and physical condition of the donor, MSCs reach replicative senescence at different passages, usually accompanied by various morphological manifestations, such as enlargement. The cell size is strongly associated with the increase of senescence associated β-galactosidase expression and actin stress fibers [39]. For example, the Wharton´s Jelly-derived MSCs reached senescence at the fourteenth passage when they became large and more elongate [12]. On the other hand, bone marrow-derived MSCs (BM-MSCs) or infrapatellar fat pad-derived MSCs were senescent already after the fifth passage and displayed irregular flattened geometry and enlarged size [13, 40]. Some authors indicated that MSCs from neonatal tissues showed no signs of cell senescence during long-term culture. Neonatal tissues exhibit certain biological properties that differ from MSCs originating from adult sources. Jin et al. demonstrated that senescence of umbilical cord blood-derived MSCs (UCB-MSCs) was slower than that of BM-MSCs or adipose tissue derived MSCs since UCB-MSCs had the highest rate of cell proliferation and clonality, and significantly lower expression of p53, p21 and p16 [41]. Non-gonadal tissues including chorion express luteinizing hormone-chorionic gonadotropin receptors (LHCG-R) which are essential for their growth during fetal development. Cismaru et. al showed beneficial effects of human chorionic gonadotropin (hCG exposure) on gene regulation in bone marrow adherent stem cells through the upregulation of pluripotency genes and selection of more primitive MSCs with a better differentiation potential [42, 43].

Telomere shortening is a well-known hallmark of cellular senescence as well as organismal aging. It acts as a counting mechanism that drives replicative senescence by limiting the mitotic potential of cells [44]. Human telomeres typically range between 10 and 15 kb [45] which corresponds to the average telomere length of early passages in our experiment. On the other hand, the late-passage telomere length of 2.88 ± 0.21 kb of our CHO-MSCs (Fig. 1D, E) was close to the range of short telomeres that represent 1–2 kb telomeric repeats that accumulate in human cell culture before senescence [46].

Senescence of CHO-MSCs at P10 was confirmed also by their transcriptional profile (Fig. 2, Suppl Fig S2). Expression of the hallmark gene of replicative senescence β-gal (GLB1) [17], the tumor suppressor p53 and cell cycle inhibitor p21 were significantly upregulated at P10, while Ki-67, a proliferation marker expressed in a cell cycle-dependent manner [47], was significantly reduced. Overexpression of the proto-oncogene c-myc in contrast to E-Ras, is not sufficient for tumorigenic transformation in MSCs without other mutations [48]. Transforming growth factor TGF-β1, 2 and 3 are involved in almost every aspect of MSCs function [49], including senescence and cell aging, which is mediated by inducing the cyclin-dependent kinase inhibitors, such as p21, and by suppressing several proliferation factors [50]. Most importantly, TGF-β together with inflammatory cytokines, is the component of the senescence-associated secretory phenotype (SASP), which is secreted in high amounts by senescent cells.

After the senescent status was defined, we focused on differences between early vs late EVs and EV-miRNA profile. EVs reflect the physiological or pathological state of parental cells and facilitate cell-to-cell communication. Nowadays they are thought to be the main carriers of the therapeutic effects previously attributed to their parental cells. The isolation and characterization of CHO-MSC-derived EVs were previously described for the first time by Janockova et al, 2021 [51]. In our study, isolated EVs of all passages were identified by EVs-typical surface markers (CD9, CD63, CD81; Fig. 3) and by markers of MSC origin (CD105, CD44, CD49e). A statistically significant overexpression of the CD81 and CD29 was observed in the late passage EVs and a significant decrease in cell-related biomarker CD133. Fu et al showed that CD81 could play a crucial role in regulating gingival fibroblast cell senescence [52]. CD29 was shown to be increased in elderly healthy individuals compared to the control young group [53]. However, the biological role of CD81 and CD29 in cell senescence has not yet been sufficiently studied. CD133 is one of the key biomarkers for isolation and characterization of stem cells and is associated with proliferative potential [54]. Its downregulation on EVs in the late passage corresponds to the loss of that potential of the parental cells.

It has been shown that with aging and senescence, production and content of EVs by MSCs may be altered, which likely impact their function and therapeutic effect [28, 55–57]. In some studies, production of EVs by MSCs was increased with aging and senescence [12, 25, 57, 58]. In another study, no difference in size between EVs of young and aged MSCs were found, however young MSCs secreted a significantly higher miRNA concentration than aged cells [59]. In our study, serial passaging did not have a significant impact on the size and amount of MSC-EVs. Even though we observed a slight increase in the size of the EVs secreted towards the late passages, this increase was not statistically significant.

Comparing early and late passage EV-miRNAs from CHO-MSCs, we found significantly altered expressions of EV-miRNAs in response to cellular senescence. Among them 16 were up-regulated and 21 down-regulated (Tables 2 and 3; Fig. 4A, B). Out of the 37 DE miRNAs, 23 were found to be associated with cellular senescence and connected pathways in KEGG pathway analysis (Suppl Tab S2). Based on the number of validated targets in the mentioned pathways, 9 miRNAs were identified as the most associated with cellular senescence (Fig. 5). miRNAs miR-145-5p, miR-16-5p, miR-20a-5p, miR-93-5p were among the most frequently identified in KEGG pathways related to cellular senescence, and it was in connection to p21 (CDKN1A), CDK4/6, CCNDs and TGFBR as their experimentally validated target genes. These miRNAs were significantly downregulated in the late passage (Fig. 5), which corresponds to the overexpression of p21 and TGF-β (Fig. 2).

MiR-145-5p is mainly considered a tumor suppressor in diverse types of cancers. Downregulation of miR-145-5p together with the downregulation of another four miRNAs identified in our study (miR-342-3p, miR-494-3p, miR-574-3p, miR-487-3p) was also found in the study of Sun et al. [60], which compared aged and young BM-MSCs-EVs. MSC-EVs containing miR-145-5p reduced inflammation in spinal cord injury by regulating the TLR4/NF-κB signaling pathway [61]. Comparison of adipose MSCs from old and young donors showed that miR-145-5p is more expressed in young MSCs [62]. More studies reported that the overexpression of miR-145-5p rejuvenated old donor or senescent adipose MSCs phenotype and augmented the functionality of these cells [62, 63]. Overall, this suggests that miR-145-5p should be present in therapeutically effective EVs. Its reduction may indicate senescence of donor MSCs or other loss-of-efficacy problems.

The most frequent overlap between previous studies of senescent cellular miRNAs of various origin and our study of senescent MSCs EV-miRNAs was the downregulation of members of the miR-17 family (in our study namely miR-20a and miR-93) [64–66]. The miRNAs of the miR-17 family, especially miR-20a and miR-93, have been found in multiple studies to decrease in senescent MSCs and were shown to target p21 [26, 65]. In our study, their experimentally validated targets were enriched mostly in pathways of cellular senescence, p53 and FoxO signaling pathways (through p21, cyclin-D1 CCND1 and TGF-β receptor TGFBR) (Suppl Tab S1, S2).

Out of all the DE miRNAs, miR-199b-5p was the most significantly downregulated in the late passage EVs and with the highest fold change (Table 3; Fig. 4A, B), however, its target proteins did not appear in senescence-related pathways. The role of this miRNA in senescence was observed in Yoo *et al,* 2014 [67], who also showed that it may control senescence indirectly through LAMC1 in BM-MSCs. The downregulation of miR-199b-5p was also detected in EVs of senescent fibroblasts [68].

MiR-335-5p was another significantly downregulated miRNA in the late passage (Figs. 4A and 5) which was directly associated with cellular senescence (Suppl Tab S1, S2). The biological role of this miRNA is somewhat contradictory. In the literature, miR-335-5p is rendered as anti-inflammatory and tumor-suppressor miRNA. For example, in the chronic rhinosinusitis mouse model, upregulation of miR-335 reduced inflammation via negative regulation of the TPX2-mediated AKT/GSK3β signaling pathway [69]. miR-335-5p also inhibited carcinoma cell proliferation, invasion, and migration. Its downregulation has an oncogenic effect [70, 71]. In BM-MSC-EVs, miR-335 downregulation impaired their functions in fracture recovery [72]. On the contrary, its expression was found to be increased with age in MSC-EVs [58, 73, 74].

From DE miRNAs, 16 were upregulated (Table 2; Fig. 4A). The miR-1307-3p was the most overexpressed miRNA in the late passage, with the highest statistical significance, however no experimentally validated targets were found for it in the mirTarbase database. Literature review revealed that miR-1307-3p has been shown to have roles in cancer, apoptosis and cell cycle arrest [75]. miR-1307–3p has low expression levels in colon adenocarcinoma tissues and cell lines, can regulate proliferation and apoptosis of colon adenocarcinoma cells via targeting ISM1 and regulating activation of Wnt3a/β-catenin signaling pathway. Overexpression of miR-1307–3p suppressed the proliferation, promoted apoptosis and arrested the cell cycle at the G1 phase, meanwhile, downregulation of ISM1 accelerated the proliferation, inhibited apoptosis and promoted cell cycle progression. The survival rate of miR-1307–3p high expression group was more than that of the low expression group [76]. miR-1307 expression is also inversely correlated with cell viability in *in vitro* model for SARS-CoV‑2 infection. miR-1307-3p was the most highly expressed miRNA in the infected cells [77].

Cellular senescence has been demonstrated to be under the regulation of miRNAs. They interact with specific mRNAs to regulate various cellular mechanisms, including cell cycle, proliferation, apoptosis and senescence. Based on previous studies, a plethora of miRNAs were implicated to play key regulatory functions in cellular senescence in MSCs. The most recent and comprehensive reviews by Potter et al (2021) and He et al (2023) [27, 28] point at their high variability from study to study, indicating the complexity of the regulation. Apart from that, their high diversity seems to be depending also on the source of MSCs, the state of the donor of the MSCs and possibly also whether they are cellular miRNAs or secreted into EVs [57]. A couple of studies observed that miRNA content secreted into EVs differed substantially from the cellular miRNA content, supporting the view that cellular miRNA and EVs mediated miRNAs are differently regulated [78, 79]. While dysregulation of cellular miRNAs in senescence has been studied for the last 15 years, the miRNA content of EVs of senescent MSCs and their potential regulatory roles is much less covered. Identifying and studying miRNAs directly related to cellular senescence may help to possibly modify/prevent replicative senescence of MSCs and their EVs *in vitro* and facilitate their prolonged therapeutic potential, which is a key requirement either for cell-based or cell-free therapy.

Multiple studies have already shown that EVs from young cells (MSCs or fibroblasts) can ameliorate senescence-related phenotype [80, 81]. However, various limitations such as low yield or insufficient therapeutic effect hamper the practical application of EVs. To overcome these limitations, engineered EVs have emerged, which provide precise targeting and efficient delivery of cargo across biological barriers [82]. Engineered EVs may deliver specific miRNAs that are downregulated in a given condition or disease and restore their posttranscriptional regulatory role [83–85]. Alternative delivery systems represent viral vectors; however, better suited for *in vitro* experiments. Such a system was successfully used in the study of Li et al, 2024, where senescent adipose-derived stem cells transfected with lentiviral vector overexpressing miR-145-5p was able to rejuvenate the cells, partly decrease senescent phenotype and increase proliferation rates [86]. A decrease in p21 expression and protein levels were also noted and the authors proposed that miR-145-5p could be used as a new target for rejuvenating old stem cells.

When we looked at the targets of DE miRNAs in the miRTarBase database, we noticed that the 21 downregulated miRNAs have 360 experimentally validated targets (Table 3), while the 16 upregulated miRNAs have only 47 targets with 7 miRNAs having no targets at all (Table 2). This suggests that the downregulated miRNAs are more frequently studied and possibly bear a higher biological importance. Restoring their expression levels by engineered delivery systems (engineered EVs, for example) might represent an approach to ameliorate cellular senescence as shown by Li et al, 2024 [86].

## Conclusion

In this work we showed that MSCs derived from chorionic tissue of young and healthy donors reached senescence at passage 10. Apart from telomere shortening, which was gradual towards the late passages, other markers of senescence, such as morphological changes or changes in gene expression profiles, appeared abruptly after the 8^th^ passage. The comparison of CHO-MSC-EVs between the early and late passages did not show statistically significant differences in their size, numbers per cells and in expression of most of the surface markers, however, we have identified significantly altered miRNAs expression in response to cellular senescence. Out of the 37 differentially expressed miRNAs, 9 miRNAs were identified as the most frequently represented in KEGG pathways directly associated with cellular senescence. The most downregulated were miRNA-199-5p, miRNA-574-3p, miR-355-5p and miRNA-145-5p and the most upregulated EV-miRNAs were miR-1307-3p, miR-3615 and miR320b. Identifying and studying miRNAs directly related to cellular senescence may help to possibly modify and prevent replicative senescence of MSCs and their EVs *in vitro* and facilitate their prolonged therapeutic potential.

## Supplementary Information

Below is the link to the electronic supplementary material.

Supplementary file1 (DOCX 674 KB)

## Data Availability

Raw sequences of miRNAs from Small RNA-Seq have been deposited to the NCBI Sequence Read Archive (SRA) database under the Bioproject accession number PRJNA1117775.
