# Supplementary material for: Effects of Replicative Senescence of Human Chorionic MSCs on their EV-miRNA Profile
Source: Stem Cell Rev Rep. 2024 Sep 21;20(8):2318–35. doi: 10.1007/s12015-024-10790-8 (PMC11554840; doi:10.1007/s12015-024-10790-8)
Supplement: Supplementary file 1 — Supplementary file1 (DOCX 674 KB) [file 12015_2024_10790_MOESM1_ESM.docx]

# **Effects of replicative senescence of human chorionic MSCs**

# **on their EVs-miRNAs profile**

# Hedviga Košuthová^1^, Lívia K. Fecskeová^1^, Jana Matejová^1^, Lucia Slovinská^1^, Marko Morávek^1^, Zuzana Bártová^2^, Denisa Harvanová^1^

^1^Associated Tissue Bank, Faculty of Medicine, Pavol Jozef Safarik University and Luis Pasteur University Hospital, Trieda SNP 1, 04011 Kosice, Slovakia

^2^Institute of Geotechnics of the Slovak Academy of Sciences, Watsonova 45, 040 01, Kosice, Slovakia

Hedviga Košuthová and Lívia Kolesár Fecskeová are co-first autors and contributed equally to this article.

Corresponding author: [livia.kolesar.fecskeova@upjs.sk](mailto:livia.kolesar.fecskeova@upjs.sk)


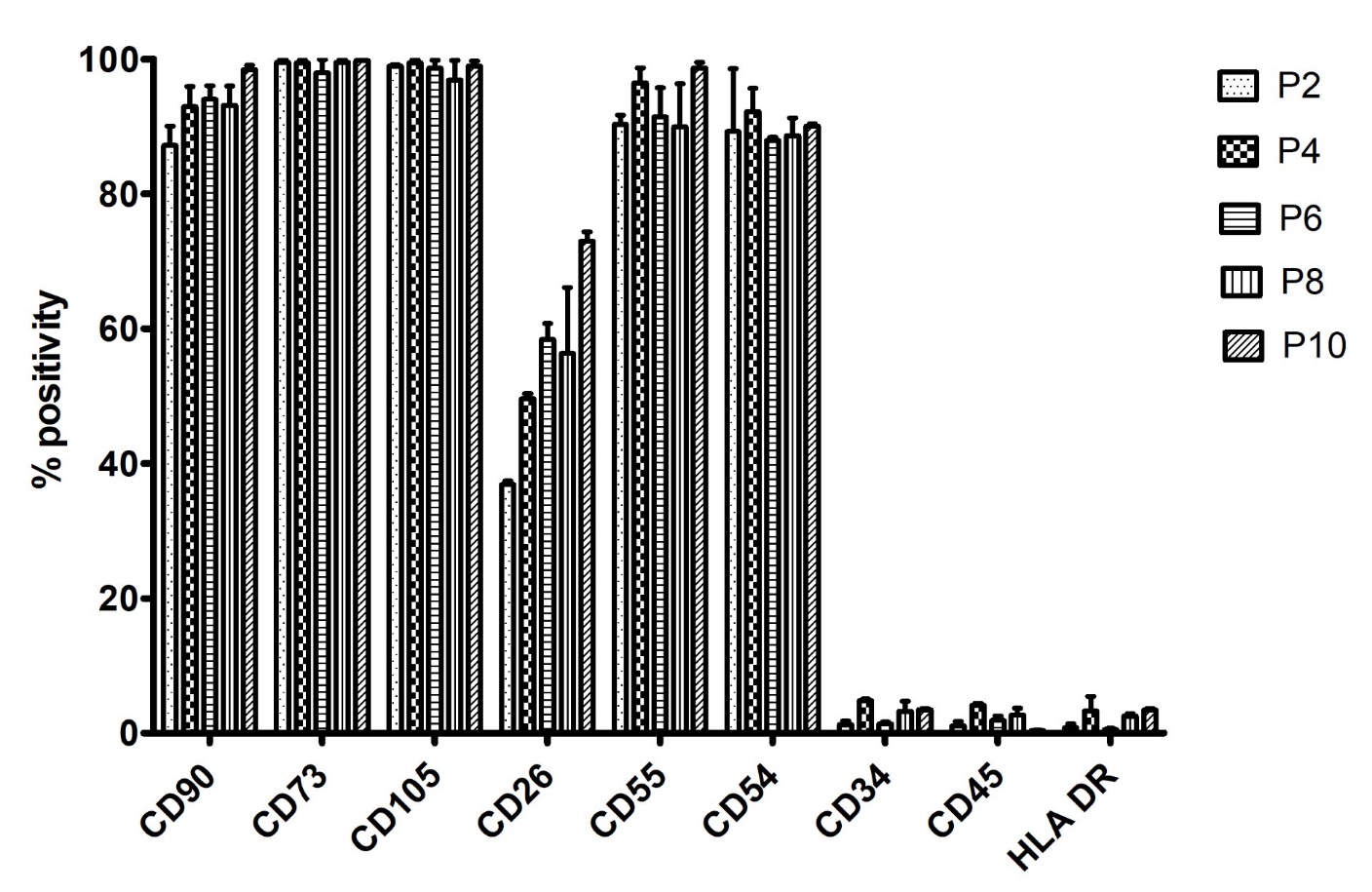


**Supplementary Figure S1.** Bar chart of flow cytometry data showing cell surface marker expression of CHo-MSCs at passages P2, P4, P6, P8 and P10, n=3

**
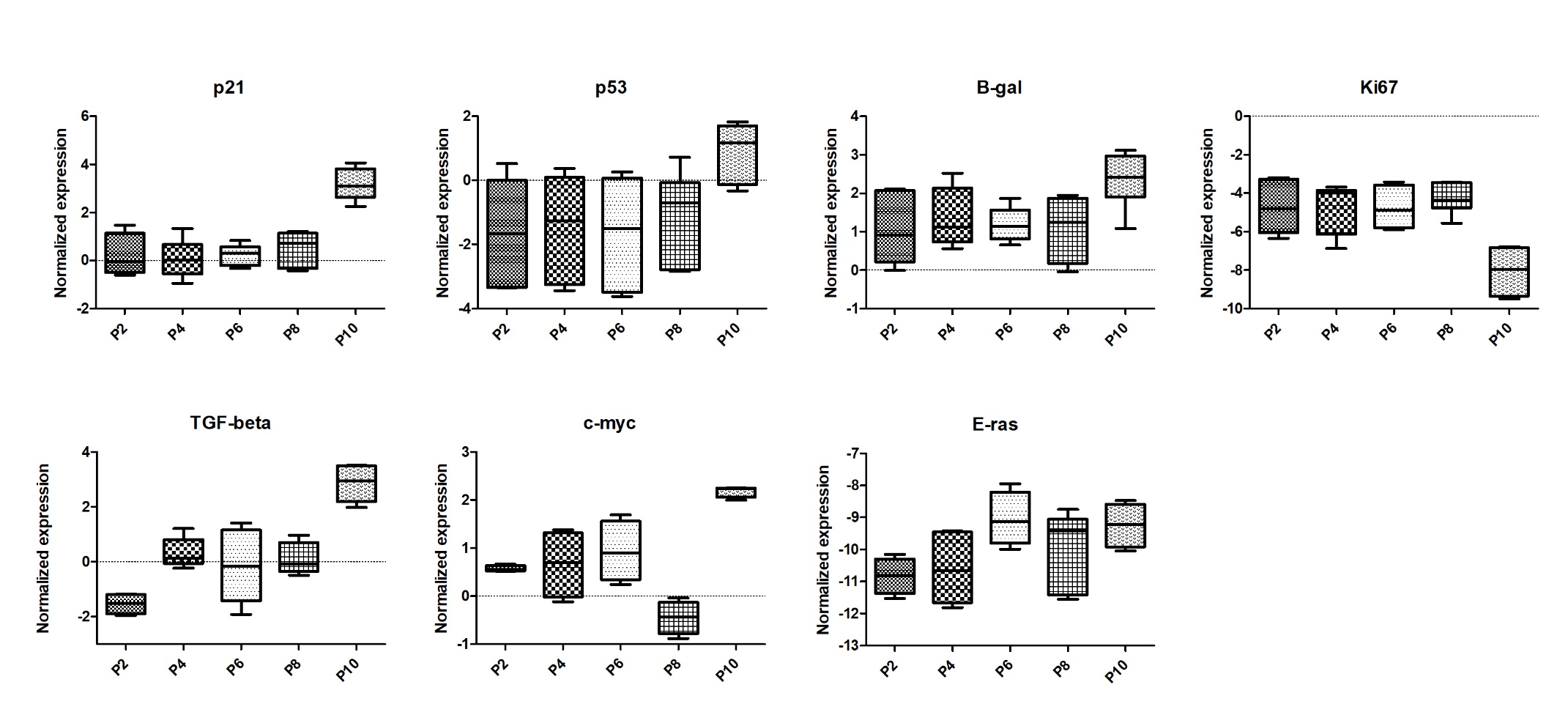
**

**Supplementary Figure S2.** Comparison of relative expression of senescence related genes p21, p53, β-gal, proliferation marker Ki67, oncogenes c-myc and E-ras and TGF-β at passages P2, P4, P6, P8 and P10 of CHo-MSCs. Gene expression is shown as normalized expression (-ΔCt, normalized Ct).

**Supplementary Table S1**: Results of KEGG pathway analysis: selected KEGG pathways related to cellular senescence, pathway fold enrichment, number of genes in each pathway and target genes present in each pathway.

| ***KEGG pathway*** | ***FDR*** | ***log10 FDR*** | ***Fold enrichment*** | ***nGenes*** | ***Pathway Genes*** | ***Genes*** |
| --- | --- | --- | --- | --- | --- | --- |
| Path:hsa04218 ***Cellular senescence*** | 1.50E-16 | 15.82 | 6.715 | 31 | 156 | CDK4 CDK6 CDKN1A CDKN2A E2F1 E2F3 AKT2 ETS1 FOXO1 FOXO3 SIRT1 MTOR KRAS SMAD2 SMAD3 MYC NFKB1 NRAS SERPINE1 PIK3CA MAPK1 MAP2K3 NA RB1 CCND1 TGFBR1 TGFBR2 CCND2 CCND3 CCNE1 CCNB2 |
| Path:hsa04115 ***p53 signaling pathway*** | 1.49E-11 | 10.83 | 8.333 | 18 | 73 | CDK4 CDK6 CDKN1A CDKN2A BBC3 IGF1 SERPINE1 NA CCND1 BCL2 BCL2L1 THBS1 PPM1D CCND2 CCND3 CCNE1 CCNG1 CCNB2 |
| Path:hsa05206 ***MicroRNAs in cancer*** | 1.01E-53 | 53.00 | 13.223 | 63 | 161 | BCL2L11 CDK6 CDKN1A CDKN1BCDKN2A PAK4 IGF2BP1 DNMT1 DNMT3A E2F1 E2F3 EGFR EP300 ERBB2 ERBB3 EZH2 FGFR3 DICER1 SIRT1MTOR FOXP1 HDAC2 HOXD10 IKBKB IRS1 ITGA5 KRAS STMN1 MCL1 MET MYC NFKB1 NOTCH1 NOTCH2 NOTCH3 NRAS ABCB1 PIK3CA PIM1 MAPK1 NA CCND1 BCL2 BCL2L2 ROCK1 BMI1 BMPR2 FSCN1 STAT3 ZEB1 THBS1 TIMP3 UBE2I VEGFA HMGA2 RECK SOCS1 CCND2 CCNE1 CCNG1 CD44 HDAC4 ZEB2 |
| Path:hsa04350 ***TGF-beta signaling pathway*** | 2.75E-07 | 6.56 | 5.451 | 15 | 93 | EP300 ID4 SMAD2 SMAD3 SMAD4 SMAD7 MYC MAPK1 NA ROCK1 BMPR2 SP1 TGFBR1 TGFBR2 THBS1 |
| Path:hsa04110  ***Cell cycle*** | 3.20E-09 | 8.49 | 5.364 | 20 | 126 | CDK4 CDK6 CDKN1A CDKN1B CDKN2A E2F1 E2F3 EP300 HDAC2 SMAD2 SMAD3 SMAD4 MYC RB1 CCND1 CCND2 CCND CCNE1 CCNB2 |
| Path:hsa04210 ***Apoptosis*** | 2.05E-09 | 8.69 | 5.218 | 21 | 136 | BCL2L11 CHUK PARP1 DFFA AKT2 FOS BBC3 BIRC5 IKBKB KRAS MCL1 MAP3K5 NA NFKB1 NRAS PIK3CA MAPK1 MAPK8 BCL2 BCL2L1 CASP2 |
| Path:hsa04151 ***PI3K-Akt signaling pathway*** | 2.86E-21 | 20.54 | 4.964 | 52 | 354 | BCL2L11 CDK4 CDK6 CDKN1A CDKN1B CHUK COL1A2 CREB1 EGFR EIF4E ERBB2 ERBB3 AKT2 FGF2 FGF10 FGFR1 FGFR3 FOXO3FN1 MTOR IFNB1 IGF1 IGF1R IKBKB IRS1 ITGA5 ITGB8 KDR KIT KRAS MCL1 MET MYC NFKB1 NRAS PIK3CA PPP2R2A MAPK1 RAC1 CCND1BCL2 BCL2L1 RXRA BDNF THBS1 VEGFA VEGFC CCND2 CCND3 CCNE1 MAGI2 |
| Path:hsa04630 ***JAK-STAT signaling pathway*** | 7.82E-09 | 8.11 | 4.589 | 22 | 162 | CDKN1A CNTFR EGFR EP300 AKT2 MTOR IFNB1 MCL1 MPL MYC PIK3CA PIM1 CCND1 BCL2 BCL2L1 STAT1 STAT3 STAT5A STAT6 SOCS1 CCND2 CCND3 |
| Path:hsa04370 ***VEGF signaling pathway*** | 7.46E-04 | 3.13 | 4.582 | 8 | 59 | AKT2 KDR KRAS NRAS PIK3CA MAPK1 RAC1 VEGFA |
| Path:hsa04068 ***FoxO signaling pathway*** | 9.23E-21 | 20.03 | 8.513 | 33 | 131 | BCL2L11 CDKN1A CDKN1B CHUK S1PR1 EGFR EP300 AKT2 FOXO1 FOXO3 SIRT1 IGF1 IGF1R IKBKB IRS1 KRAS SMAD3 SMAD4 NRAS PIK3CA MAPK1 MAPK8 NA CCND1 BNIP3 SOD2 BRAF STAT3 TGFBR1 TGFBR2 CCND2 CCNB2 |
| Path:hsa04150 ***mTOR signaling pathway*** | 9.61E-08 | 7.02 | 4.360 | 20 | 155 | CHUK EIF4E AKT2 MTOR IGF1 IGF1R IKBKB IRS1 KRAS NRAS PIK3CA WNT4 MAPK1 NA BRAF WNT1 WNT5A WNT2B FZD7 |
| Path:hsa04010 MAPK signaling pathway | 6.59E-14 | 13.18 | 4.368 | 38 | 294 | MAP3K2 CHUK DUSP2 EGFR ERBB2 ERBB3 AKT2 FGF2 FGF10 FGFR1 FGFR3 FOS IGF1 IGF1R IKBKB KDR KIT KRAS STMN1 MEF2C MAP3K5 MET MYC NF1 NFKB1 NRAS MAPK1 MAPK8 MAP2K3 RAC1 RASA1 BDNF BRAF TGFBR1 TGFBR2 NA VEGFA VEGFC |

**Supplementary Table S2.** List of 23 miRNAs that have experimentally validated targets in selected pathways related to cellular senescence. Numbers of miRNA targets in each pathway is shown per miRNA and per pathway. Pathway fold enrichment and statistical strength is shown based on KEGG analysis. CS – cellular senescence; SP – signaling pathway.

|  |  |  | **Number of experimentally validated miRNA targets in each pathway**  **(based on miRTarBase database)** | | | | | | | | | | | |
| --- | --- | --- | --- | --- | --- | --- | --- | --- | --- | --- | --- | --- | --- | --- |
|  | KEGG pathway analysis |  | ***PI3K-Akt SP*** | ***FoxO***  ***SP*** | ***p53 SP*** | ***CS*** | ***MAPK SP*** | ***Apoptosis*** | ***Cell cycle*** | ***JAK-STAT SP*** | ***mTOR SP*** | ***TGF-beta SP*** | ***VEGF***  ***SP*** | ***Total targets***  ***in all pathways/miRNA*** |
|  |  | **Pathway fold enrichment** | 4.96 | 8.51 | 8.33 | 6.72 | 4.37 | 5.22 | 5.36 | 4.59 | 4.36 | 5.45 | 4.58 |  |
|  |  | **Pathway log10 FDR** | 20.54 | 20.03 | 10.82 | 15.82 | 13.18 | 8.68 | 8.49 | 8.10 | 7.01 | 6.56 | 3.12 |  |
| miRNAs differential expression in late vs early passage | | |  |  |  |  |  |  |  |  |  |  |  |  |
| **miRNA** | ***log2FoldChange*** | ***p-adj*** |  |  |  |  |  |  |  |  |  |  |  |  |
| ***hsa-mir-145-5p*** | -2.615 | 3.10E-06 | 13 | 10 | 5 | 10 | 8 | 2 | 7 | 4 | 6 | 5 | 2 | 72 |
| ***hsa-mir-16-5p*** | -1.585 | 2.84E-02 | 13 | 4 | 7 | 5 | 7 | 2 | 5 | 4 | 2 | 0 | 2 | 51 |
| ***hsa-mir-20a-5p*** | -1.725 | 2.98E-02 | 5 | 4 | 2 | 4 | 5 | 2 | 3 | 4 | 0 | 4 | 0 | 33 |
| ***hsa-let-7g-5p*** | -1.621 | 1.39E-02 | 5 | 2 | 3 | 5 | 3 | 2 | 3 | 3 | 1 | 4 | 1 | 32 |
| ***hsa-mir-214-3p*** | -2.508 | 5.67E-04 | 6 | 4 | 2 | 3 | 6 | 5 | 1 | 1 | 2 | 0 | 2 | 32 |
| ***hsa-mir-1-3p*** | -3.223 | 1.01E-04 | 6 | 3 | 1 | 3 | 4 | 2 | 0 | 3 | 5 | 0 | 3 | 30 |
| ***hsa-mir-9-5p*** | -3.597 | 3.38E-02 | 4 | 5 | 1 | 5 | 4 | 2 | 0 | 0 | 0 | 1 | 0 | 22 |
| ***hsa-mir-335-5p*** | -4.839 | 6.39E-05 | 2 | 2 | 1 | 3 | 3 | 3 | 2 | 0 | 1 | 2 | 1 | 20 |
| ***hsa-mir-574-3p*** | -2.976 | 9.61E-07 | 4 | 4 | 0 | 1 | 2 | 0 | 3 | 3 | 0 | 2 | 1 | 20 |
| ***hsa-mir-222-3p*** | 1.633 | 1.11E-02 | 4 | 3 | 1 | 2 | 2 | 2 | 1 | 2 | 0 | 0 | 0 | 17 |
| ***hsa-mir-487b-3p*** | -1.812 | 7.65E-03 | 3 | 1 | 0 | 2 | 2 | 1 | 1 | 1 | 2 | 1 | 1 | 15 |
| ***hsa-mir-494-3p*** | -2.583 | 6.25E-06 | 3 | 1 | 1 | 2 | 1 | 1 | 3 | 1 | 0 | 1 | 0 | 14 |
| ***hsa-mir-93-5p*** | -2.260 | 9.08E-04 | 2 | 2 | 1 | 2 | 2 | 0 | 1 | 1 | 0 | 2 | 0 | 13 |
| ***hsa-mir-342-3p*** | -1.920 | 2.31E-02 | 2 | 1 | 0 | 2 | 1 | 1 | 0 | 1 | 2 | 2 | 1 | 13 |
| ***hsa-mir-152-3p*** | -1.318 | 1.39E-02 | 3 | 1 | 1 | 1 | 1 | 0 | 1 | 1 | 1 | 0 | 0 | 10 |
| ***hsa-mir-130a-3p*** | -1.771 | 1.08E-02 | 1 | 1 | 1 | 0 | 1 | 0 | 0 | 0 | 1 | 2 | 0 | 7 |
| ***hsa-mir-320b*** | 2.434 | 1.03E-04 | 1 | 0 | 0 | 1 | 1 | 0 | 1 | 1 | 0 | 0 | 0 | 5 |
| ***hsa-mir-452-5p*** | -1.873 | 1.42E-02 | 1 | 1 | 0 | 0 | 1 | 0 | 1 | 0 | 0 | 0 | 1 | 5 |
| ***hsa-mir-361-5p*** | -1.748 | 3.39E-02 | 1 | 0 | 0 | 0 | 1 | 0 | 0 | 1 | 0 | 0 | 1 | 4 |
| ***hsa-mir-708-5p*** | -2.258 | 1.43E-03 | 0 | 0 | 0 | 0 | 0 | 2 | 0 | 1 | 0 | 0 | 0 | 3 |
| ***hsa-mir-199b-5p*** | -4.480 | 2.74E-09 | 1 | 0 | 0 | 0 | 1 | 0 | 0 | 0 | 0 | 0 | 0 | 2 |
| ***hsa-mir-374b-5p*** | -2.382 | 1.08E-02 | 0 | 0 | 0 | 0 | 1 | 0 | 0 | 0 | 0 | 0 | 1 | 2 |
| ***hsa-mir-125b-1-3p*** | 1.766 | 1.35E-03 | 0 | 1 | 0 | 0 | 0 | 0 | 0 | 0 | 0 | 0 | 0 | 1 |
